# Advancing Defect Detection in Laser Welding: A Machine Learning Approach Based on Spatter Feature Analysis

**DOI:** 10.3390/s26061825

**Published:** 2026-03-13

**Authors:** Gleb Solovev, Evgenii Klokov, Dmitrii Krasnov, Mikhail Sokolov

**Affiliations:** 1 AI Institute, ITMO University, Kronverksky Prospekt 49, Saint Petersburg 197101, Russia; gvsolovev@itmo.ru (G.S.); dmitrii.krasnov@itmo.ru (D.K.); mikhail.sokolov@itmo.ru (M.S.); 2 Institute of Laser Technologies, ITMO University, Kronverksky Prospekt 49, Saint Petersburg 197101, Russia

**Keywords:** machine learning, neural networks, quality assurance, signal processing, laser beam welding

## Abstract

Full-penetration laser welding (FPLW) is increasingly adopted in manufacturing pipelines, yet its industrial scalability is constrained by in-process defect formation, particularly incomplete penetration. To address this, we propose a sensor-driven framework for non-destructive monitoring and automated defect detection that uses infrared (IR) thermography as the primary in situ sensing modality and applies deep learning to the acquired thermal signals. High-speed IR camera recordings were processed to track spatter and the weld zone, yielding a time series of physically interpretable spatiotemporal features (mean spatter area, mean spatter temperature, number of spatters, and mean welding zone temperature). Defect recognition is formulated as a multi-label classification problem targeting incomplete penetration, sagging, shrinkage groove, and linear misalignment, and multiple temporal models were evaluated on the same sensor-derived feature sequences. Experimental validation on 09G2S pipeline steel demonstrates that the proposed time series pipeline based on a hybrid CNN–transformer achieves a mean Average Precision (mAP) of 0.85 while preserving near-real-time inference on a CPU. The results indicate that IR thermography-based spatter dynamics provide actionable sensing signatures for automated defect prediction and can serve as a foundation for closed-loop quality control in industrial laser pipeline welding.

## 1. Introduction

Laser Beam Welding (LBW) is widely employed in numerous industrial sectors due to its high precision, deep penetration capabilities, and overall process efficiency. Considerable research efforts have been devoted to further optimising this technique, as LBW is increasingly recognized as one of the most promising joining methods available. This is largely attributable to several distinctive advantages: (1) the non-contact nature of the process; (2) the ability to perform single-side joining; and (3) the exceptionally high energy density of the laser beam, which enables the formation of a weld within a fraction of a second. These characteristics contribute to a reduced heat-affected zone and, consequently, minimal deformation and residual stresses [1].

In addition to these advantages, LBW provides reduced heat input, minimal thermal distortion, and improved joint integrity compared with conventional welding techniques. These attributes make it particularly suitable for applications that demand high-quality and highly reliable welds [2]. Among the various laser welding configurations, full-penetration laser welding is often considered a favourable option due to its capacity to produce single-pass welds, thereby increasing productivity and reducing operational costs [3].

In sectors such as oil and gas, energy, and water distribution, pipeline welding is critical to ensuring both the structural integrity and the long-term operational reliability of pipeline systems. Historically, pipeline fabrication has relied on arc-based techniques—including gas metal arc welding and submerged arc welding (SAW)—which typically require joint grooving, multiple welding passes, and extensive post-weld inspection procedures [4]. Although these methods have proven effective, they remain time-consuming and labour-intensive. By contrast, FPLW significantly reduces filler metal consumption—by up to 90% compared with SAW—and offers welding speeds that are three to five times higher [5].

Despite the well-documented advantages of laser welding, its widespread adoption in pipeline manufacturing and maintenance is still limited by several technical challenges. A major concern is the formation of weld defects—such as spattering, porosity, and keyhole instability—which may compromise joint strength and long-term performance [6]. Among these, spattering is considered a particularly critical phenomenon, as it can lead to contamination of the weld seam, material loss, and defects such as undercutting and inclusions [7].

Although a substantial body of research has examined the influence of welding parameters on spatter dynamics [8], there is still a gap in the development of real-time defect detection methods that will lead to enabling process control and continuous weld quality monitoring. Recent studies have investigated a range of factors affecting spatter generation, including keyhole behaviour, evaporation-driven recoil pressure, and melt pool dynamics. Correlations between spatter characteristics and key process parameters—such as laser power, as was shown by Cheng et al. [9] and Zoe et al. [10]; welding speed; and shielding gas conditions—have been well established in the laser welding literature, where variations in these parameters are shown to significantly influence spatter generation, morphology, trajectory, and thermal behaviour. Zhang et al. [11] analysed the influence of penetration mode and beam configuration on spatter network formation, and Wang et al. [12] clarified the dependence of oscillation parameters and heat input conditions on spatter behaviour. While these investigations have yielded valuable insights into the underlying physical mechanisms, there remains a need for advanced, data-driven techniques capable of extracting quantitative spatter features to support real-time prediction of defect formation.

Pipeline materials, particularly modern high-strength steels used in oil, gas, and hydrogen pipeline transportation, are explicitly designed with weldability as a critical engineering consideration. However, the problem of controllable laser penetration depth and other common laser welding defects remains a challenge [13].

In the FPLW of pipeline steels, defects such as porosity and cracking are relatively less prevalent due to the high weldability of these materials and their optimisation for joining processes. However, incomplete penetration or lack of fusion remain significant and comparatively common defects. These defects typically arise from suboptimal process conditions, including insufficient heat input, weld misalignment, or improper focal positioning, and can markedly compromise the structural integrity of the welded joint [14,15]. Addressing these issues requires a comprehensive understanding of the interplay between welding parameters and defect formation, as well as the development of advanced real-time monitoring and control systems to ensure consistent weld quality.

A variety of Weld Penetration Depth Control (WPDC) methods have been proposed in the literature, most of which rely on indirect process signals or require specific modifications to the welding setup. One of the earliest closed-loop WPDC approaches involved plasma analysis. Sibillano et al. [16] demonstrated that real-time correlation between electron temperature measured via spectrometry and the reference penetration depth in ${\mathrm{CO}}_{2}$ laser welding could be used for control purposes. However, this method has a notable limitation—it requires precise calibration for each welding configuration, material, and desired penetration depth, thereby restricting its industrial flexibility. Harooni et al. [17] proposed a similar strategy based on correlating plasma ionisation temperature with penetration depth. Yet, as noted by You et al. [18], fluctuations in ionisation temperature can themselves indicate weld imperfections, which constrains the suitability of this approach for WPDC in industrial environments.

Other in-process monitoring and control techniques have leveraged photodiodes to collect signals such as thermal radiation, ultraviolet emissions, laser back-reflection, and visible light from the weld zone [19].

Acoustic signal processing offers significant potential for extracting meaningful information from the laser welding process. To better understand the relationship between optical emissions and airborne acoustic responses, Hoffman et al. [20] conducted a combined spectroscopic and acoustic analysis, demonstrating that characteristic audio frequencies depend on both the material type and its thickness. Further evidence was provided by Yusof et al. [21], who concluded that acoustic methods are suitable for online monitoring and control of weld penetration depth. However, the authors also highlighted that the inherent sensitivity of acoustic measurements to ambient noise presents a major limitation for their robust deployment in industrial environments. Complementary findings were reported by Fischer et al. [22], who showed, through experiments on glass and metal sheets, that high-frequency airborne ultrasound constitutes a potent technique for weld quality assessment and WPDC. This conclusion is supported by Authier et al. [23], who demonstrated that acoustic analysis can be further enhanced when combined synergistically with other sensing modalities.

As an alternative line of development, the transition from conventional LBW to hybrid welding processes expands the range of feasible monitoring and control strategies. For example, Bîrdeanu et al. [24] investigated WPDC in a hybrid welding configuration and demonstrated that the frequency of the TIG arc can be used as a reliable indicator of weld penetration depth. While relevant to the broader field of process control, a comprehensive review of hybrid or non-remote laser welding techniques lies outside the scope of the present study.

Despite their diversity, the methods described above share a common set of challenges, most notably the need for extensive databases of reference signals collected under tightly controlled conditions. Although these approaches remain promising—especially when viewed in light of recent advances in machine learning and artificial intelligence—the exclusive reliance on data-driven techniques is insufficient for industrial implementation. The inherent variability of real-world laser welding processes, including material composition, thickness, laser characteristics, joint configuration, and gap conditions, cannot be reliably captured by data alone. As noted in [25], effective WPDC solutions for industry require approaches that can accommodate these variations while maintaining robustness, adaptability, and real-time operability.

Analysing various methods for in-process quality control during laser welding, You et al. [18] suggested that the most reliable direct WPDC method remains X-ray imaging. However, the practical challenges associated with implementing X-ray systems in industrial environments—particularly concerning safety requirements, equipment complexity, and integration constraints—significantly restrict their applicability [26]. In their comprehensive investigation of weld penetration depth monitoring techniques, Bautze et al. [27] concluded that, aside from X-ray imaging, the only method capable of providing a direct, in-process measurement of keyhole depth is Optical Coherence Tomography.

Within the context of real-time defect detection in FPLW, the selection of sensing technology plays a decisive role in achieving accurate monitoring of process stability and weld quality. A wide range of sensors has been explored for weld diagnostics; however, infrared cameras offer advantages due to their ability to capture thermal emissions from the weld pool and from spatter dynamics. In contrast to visible-range imaging systems, which may be affected by process-related obstructions such as fumes, vapour plumes, or plasma, IR imaging provides a robust and contactless means of visualising temperature fluctuations and identifying thermal anomalies that are potentially associated with weld defects. Furthermore, IR cameras enable high-speed acquisition of spatter behaviour, allowing for the extraction of discriminative features such as spatter temperature, trajectory, and cooling rate—parameters that are examined in this study for their correlation with weld quality. By integrating IR camera-based data acquisition with machine learning-enabled analysis, the present work aims to advance defect prediction capabilities and ultimately contribute to improved process monitoring in FPLW.

Recent advancements in machine learning (ML) and computer vision have opened new possibilities for data-driven weld quality assessment. Various researchers have utilized automated vision systems to extract quantitative spatter metrics; for instance, Schweier et al. [28] developed a machine vision approach using Kalman filter-based tracking to identify spatter formation mechanisms during beam oscillation. Huang et al. [29] employed motion tracking to statistically analyze spatter ejection directions and velocities, linking them to vapor plume pressure. Furthermore, Hartung et al. [30] demonstrated the efficacy of deep learning-based semantic segmentation for real-time spatter detection and its use as a signal for defective seams. Given the stringent safety and durability requirements of pipeline infrastructure, integrating ML-based spatter analysis into defect detection frameworks could substantially enhance weld reliability while minimizing reliance on post-weld inspections.

A diverse range of ML techniques has been employed to establish relationships between sensor-derived features and defect occurrences during laser welding [31]. Classical ML methods, such as decision tree (DT), linear regression, random forest (RF), logistic regression (LR), gradient boosting (GB), and support vector machine (SVM), remain widely adopted in industrial applications due to their interpretability, relatively low computational cost, and robustness in structured data environments. For instance, Xiao et al. [32] utilized linear regression to predict welding depth using coaxial pyrometer data. Chianese et al. [33] applied DT, RF, and SVM to classify weld defects (e.g., overpenetration, lack of fusion) based on photodiode signal features. Zhao et al. [34] demonstrated the efficacy of RF models in monitoring weld penetration via laser keyhole imaging. Paulson et al. [35] employed LR and GB to link thermal history with keyhole porosity in laser powder bed fusion.

Beyond these classical approaches, modern deep learning (DL) methods offer advanced capabilities in handling complex, high-dimensional data. Multilayer perceptrons (MLPs) and convolutional neural networks (CNNs) have demonstrated promise in welding defect analysis [36]. Gao et al. [37] successfully achieved multi-class classification of five weld defect types using keyhole images and an MLP framework. Zhang et al. [38] implemented a CNN to monitor weld penetration states directly from raw pixel data captured by coaxial vision systems. These advancements underscore the potential of ML and DL techniques in enabling automated, high-precision weld quality assessment.

In addition to these domain-specific developments, it is instructive to consider methodologies established in broader fields such as video classification and time series analysis, as the task addressed in this study is fundamentally aligned with the classification of dynamic spatiotemporal patterns. Spatter behaviour, as captured by IR camera imaging, exhibits evolution in both space and time. As such, the analysis may leverage either (i) embeddings extracted directly from raw pixel sequences or (ii) engineered features organised into time series representations. These perspectives provide a conceptual bridge between welding-specific monitoring approaches and state-of-the-art ML architectures developed for sequence modelling and temporal pattern recognition, forming a foundation for the approach proposed in this work.

In the field of video classification, widely adopted baseline architectures typically rely on convolutional neural networks employing either 2D or 3D convolutional layers. For example, Hara et al. [39] introduced a three-dimensional extension of the ResNet architecture, demonstrating that 3D convolutions substantially improve video classification accuracy by explicitly capturing spatiotemporal dependencies. Nevertheless, studies such as [39,40] also acknowledge that 2D convolutional models remain capable of performing video classification tasks, albeit with reduced accuracy, despite their inherent limitation in modelling temporal evolution directly. These observations provide valuable context for selecting suitable architectures for analysing spatter dynamics in infrared video data, where both spatial structure and temporal progression are essential for reliable defect detection.

For time series analysis, the task involves processing sequences of one-dimensional signals. A classical approach is to construct a feature vector based on aggregated statistics (e.g., mean, standard deviation, skewness, or kurtosis) within a sliding window, followed by classification using basic ML or DL models. Currently, 1D CNNs [41], transformers [42], recurrent networks with Long Short-Term Memory (LSTM) cells [43], and hybrid architectures combining convolutional and transformer layers [44] are widely used for this purpose.

1. Characterize the relationship between spatter dynamics and weld defects in laser welding.
2. Develop a video processing framework for extracting spatiotemporal features from spatter dynamics.
3. Implement and evaluate a deep learning pipeline for classifying weld quality based on spatter behavior.
4. Validate the proposed methodology through experimental pipeline steel laser welding trials and assess its effectiveness in defect prediction and process optimization.

The innovation of this work lies in systematically defining a multi-dimensional spatiotemporal spatter feature set—including mean spatter area, mean spatter temperature, number of spatters, and mean welding zone temperature—specifically for 09G2S pipeline steel, and for the first time evaluating the correlation between these features and various defects to achieve a real-time classification model. Notably, the proposed method operates on a non-specialized thermal camera, providing a cost-effective and accessible alternative to expensive proprietary monitoring systems from vendors such as Precitec, Trumpf, or IPG.

## 2. Materials and Methods

### 2.1. Laser Welding Setup

The experiments were conducted using 09G2S structural pipeline steel samples of dimensions 3 × 150 × 450 mm. The chemical composition and mechanical properties of the steel are given in Table 1.

**Figure 1:**
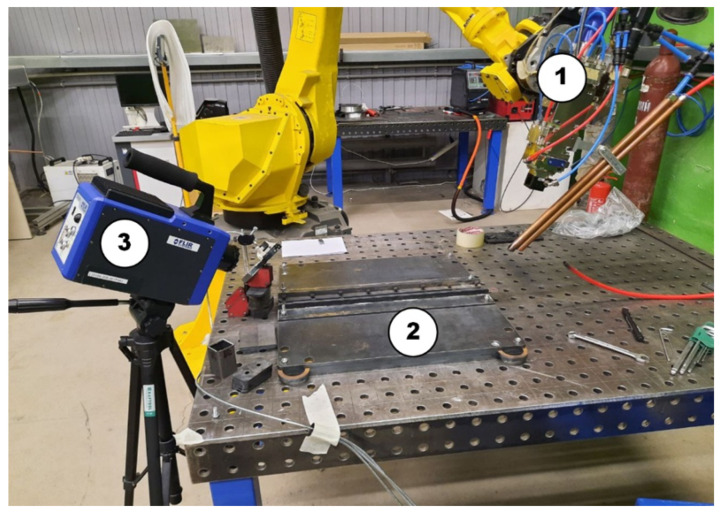
Experimental setup: (1) a WSX ND40 welding head with shielding gas pipes; (2) steel samples with clamping system; and (3) a FLIR Systems Titanium 520 M Camera (FLIR Systems, Inc., Wilsonville, OH, USA) installed at a distance of 50 cm from the weld zone.

Materials were welded by continuous wave multi-mode MAXPhotonics MFSC-3000 (Maxphotonics Co., Ltd., Shenzhen, China) in a butt joint welding setup. All experiments were performed with Argon shielding gas at a 20 L/min flowrate and without filler wire. The welding setup is shown in Figure 1 and equipment specification is shown in Table 2. The samples were mechanically cleaned before welding to remove rust and other possible weld contaminants.

**Figure 2:**
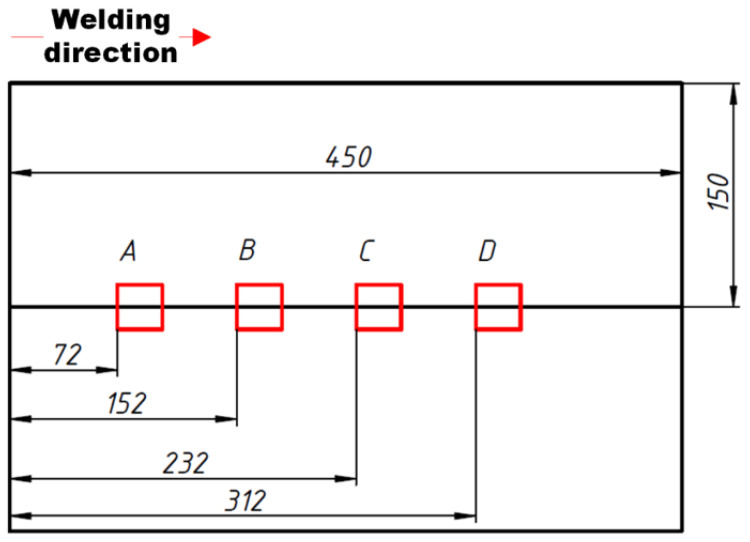
Sample cut locations.

After welding, the samples were cut at four places: 72, 152, 232, and 312 mm from the weld start, marked with letters (A, B, C, and D) as shown in Figure 2. Once the samples were extracted and polished, they were etched in a 4% Nital solution and the cross-sections were photographed using a LOMO MSP-1 microscope (LOMO (Leningrad Optical Mechanical Association), Saint Petersburg, Russia). The geometry of the weld and the weld defects were recognized on the cross-section photographs.

### 2.2. Design of Experiments

The experiments were focused on investigation of the ability to detect weld defects with an ML approach and analysis of the welding process parameters on the selected laser welding defects. A multi-level factorial design was employed, varying two key parameters—laser power ($P_{L}$) and welding speed ($V_{w}$)—at three levels each. Other parameters—the focal position ($F_{z}$) relative to the sample surface and the inclination angle (α) of the laser head—were maintained at fixed values. The experimental plan included a minimum of three replication points per condition, resulting in a total of 34 welding trials. From these trials, 136 macrosections were extracted and analysed. The experimental parameters, factors, and levels are summarized in Table 3. Analysis of these macrosections revealed several weld defects. The types, corresponding standards, frequencies, and visual examples of these defects are detailed in Table 4.

### 2.3. Feature Extraction from Spatter

Detecting spatters during laser welding is complicated by interfering radiation from the metal vapor plume and the background thermal emission of the workpiece. To reduce this interference, a background suppression filter is applied prior to spatter tracking. This filter enhances spatter-related radiation while attenuating the general background heat. The algorithm employs directional Laplacian-of-Gaussian (LoG) filtering combined with a minimum selection method as proposed by Kim [47]. Equation (1) show the overall process of generating directional (east, west, south, and north) LoG kernels, where (x,y) denotes a pixel position, σ is the standard deviation of the LoG kernel, and *N* is the kernel size (N=9 and σ=1.8 in our experiments). Equation (2) show the process of extracting directional feature maps with the generated LoG kernels via directional convolution. The final feature map was obtained as the minimum of the directional feature maps at each pixel position.

(1) $$\begin{array}{c} f_{E}\left(x\right)=\frac{1}{\sqrt{2\pi }\sigma^{3}}\left(1-\frac{x^{2}}{\sigma^{2}}\right)\exp\left(-\frac{x^{2}}{2\sigma^{2}}\right)\\ \mathrm{where}\;x=0,1,2,\ldots ,N\\ f_{W}\left(x\right)=\frac{1}{\sqrt{2\pi }\sigma^{3}}\left(1-\frac{x^{2}}{\sigma^{2}}\right)\exp\left(-\frac{x^{2}}{2\sigma^{2}}\right)\\ \mathrm{where}\;x=0,-1,-2,\ldots ,-N\\ f_{S}\left(y\right)=\frac{1}{\sqrt{2\pi }\sigma^{3}}\left(1-\frac{y^{2}}{\sigma^{2}}\right)\exp\left(-\frac{y^{2}}{2\sigma^{2}}\right)\\ \mathrm{where}\;y=0,1,2,\ldots ,N\\ f_{N}\left(y\right)=\frac{1}{\sqrt{2\pi }\sigma^{3}}\left(1-\frac{y^{2}}{\sigma^{2}}\right)\exp\left(-\frac{y^{2}}{2\sigma^{2}}\right)\\ \mathrm{where}\;y=0,-1,-2,\ldots ,-N \end{array}$$

(2) $$\begin{array}{c} I_{E}\left(x,y\right)=I\left(x,y\right)⊗f_{E}\left(x\right)\\ I_{W}\left(x,y\right)=I\left(x,y\right)⊗f_{W}\left(x\right)\\ I_{S}\left(x,y\right)=I\left(x,y\right)⊗f_{S}\left(y\right)\\ I_{N}\left(x,y\right)=I\left(x,y\right)⊗f_{N}\left(y\right) \end{array}$$

**Figure 3:**
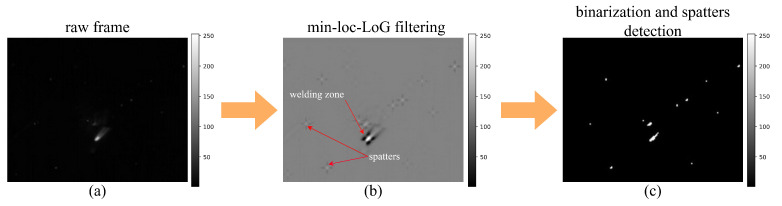
Image processing pipeline: (**a**) raw frame; (**b**) filtered feature map; and (**c**) binarized feature map.

This combination effectively removes false detections near the vapor plume edges and improves spatter visibility. Representative examples of the raw and filtered image frames are shown in Figure 3a and Figure 3b, respectively. Following preprocessing, the filtered frame is binarized using a constant threshold to produce a binary mask that highlights both detected spatters and the welding zone, as illustrated in Figure 3c. This binary mask is subsequently segmented into individual spatter regions, from which spatial coordinates and size parameters are extracted. Using the same coordinates, corresponding intensity values (which reflect temperature) are sampled from the original thermal data. For each frame, the following four spatiotemporal features are computed:

- Mean spatter area [spatter area]—the average area of spatter bounding boxes;
- Mean spatter temperature [spatter temp]—the average digital level from the FLIR camera pixels within spatter regions;
- Number of spatters per frame [number of spatters]—the total count of detected spatter regions;
- Mean welding zone temperature [welding zone temp]—the average digital level within the detected weld pool region.

**Figure 4:**
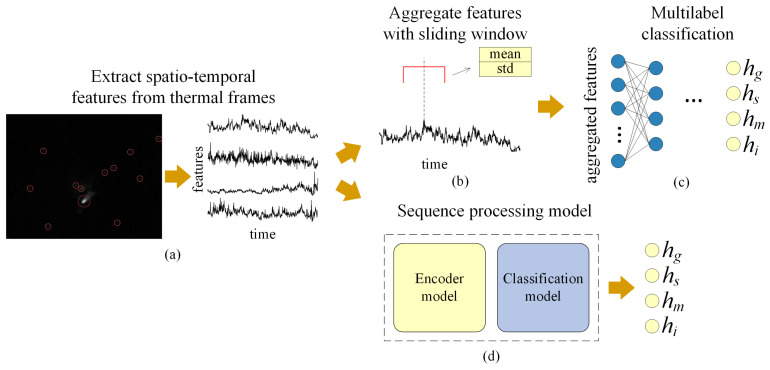
Presented defect prediction pipeline: (**a**) feature extraction; (**b**) feature aggregation; (**c**) classification with aggregated features; and (**d**) fixed-length sequence processing.

These features were designed to encapsulate both the dynamic behaviour and the thermal characteristics of the welding process, thereby enabling the identification of patterns associated with weld quality and defect formation. The extracted features were computed for each frame, producing a continuous time series of spatiotemporal descriptors (Figure 4a).

### 2.4. Deep Learning Approach

We formulated the defect classification task as a multilabel problem with four target classes: shrinkage groove ($h_{g}$), sagging ($h_{s}$), linear misalignment ($h_{m}$), and incomplete penetration ($h_{i}$). Multiple defects can co-occur within a single sample, and therefore we developed two distinct approaches to classify them based on extracted spatiotemporal features. These approaches correspond to different data preprocessing strategies, illustrated in Figure 4b,d.

**Figure 5:**
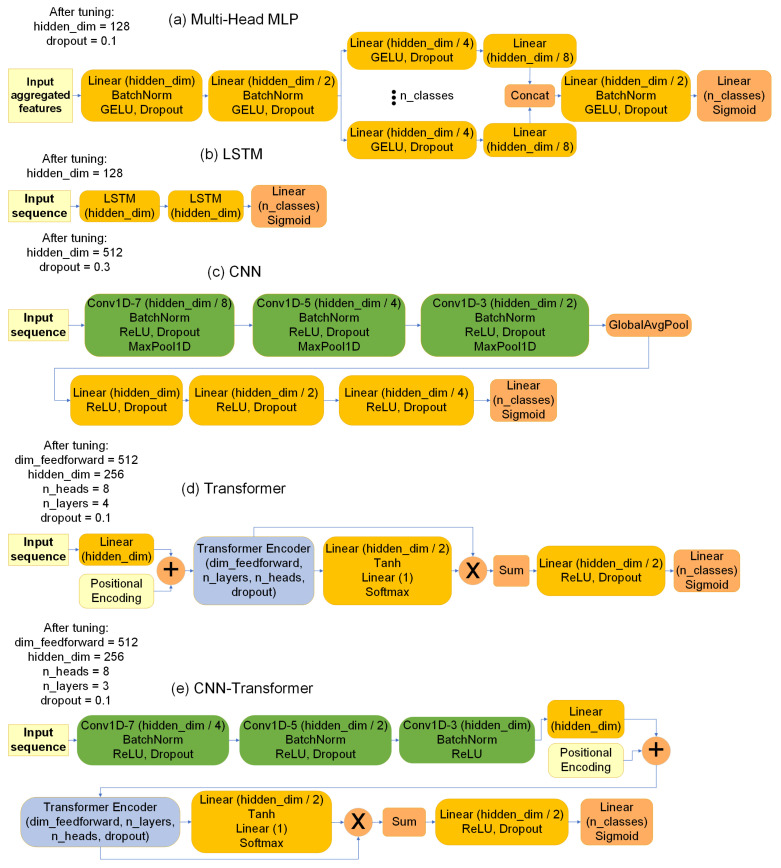
Architectures of the networks under comparison: (**a**) multi-head MLP; (**b**) LSTM; (**c**) CNN; (**d**) transformer; and (**e**) CNN–transformer.

The first approach aggregated per-frame feature time series within a sliding temporal window. For each window, the mean and standard deviation of the four extracted features were computed, yielding a fixed-dimensional representation (Figure 4b). Window size was optimized as a key hyperparameter to maximize classification performance. A custom MLP architecture with parallel class-specific heads classified these aggregated feature vectors (Figure 5a). The individual head outputs were concatenated and passed through a final shared linear layer to generate multi-label predictions (Figure 4c).

The second approach processed fixed-length sequences of per-frame features directly, without temporal aggregation (Figure 4d). A suite of sequence processing models was developed and evaluated, each comprising a temporal encoder for feature extraction followed by a linear classifier for final prediction.

Four encoder architectures were implemented: convolutional neural networks, long short-term memory networks, transformers, and a hybrid CNN–transformer architecture (Figure 5). Each encoder operated on identical input feature sequences, enabling direct comparison of temporal modelling capabilities. Key hyperparameters were systematically tuned across all models to optimize evaluation metrics, with the final network architectures illustrated in Figure 5.

Model performance was evaluated using stratified four-fold cross-validation with video-wise splits to prevent data leakage. Since each welding trial corresponds to a unique thermogram, partitioning was performed at the video level to ensure that all temporal segments derived from a single physical weld were assigned exclusively to the same fold. This procedure eliminates any correlation between training and validation sets at the weld level.

Stratification was performed based on the presence or absence of incomplete penetration ($h_{i}$) defects in each video in order to preserve class distribution across folds and mitigate class imbalance during training and validation. Notably, different defect types exhibit significant correlations; the presence of a given defect is often associated with the occurrence of other defect types. This correlation reinforces the need for stratification to ensure representative sampling of all defect combinations across folds. Alternative imbalance-handling techniques, including Focal Loss and resampling, were explored in preliminary experiments but did not yield measurable improvements, and were therefore not adopted. We attribute this to the fact that the engineered feature set already provides high class separability for the majority of samples, and the introduction of such techniques risked over-emphasizing rare noise patterns or outliers in the minority classes.

Temporal data were extracted with a 50% overlap, employing sliding windows in the first scheme and fixed-length sequences in the second. Preliminary experiments demonstrated that Z-score and Min–Max normalization degraded performance; consequently, raw feature values were used as direct model input. Normalization reduced classification accuracy, as it removed the absolute physical magnitudes that encode meaningful distinctions between defect types and welding conditions. Since the four descriptors are defined in physically meaningful units and already operate on a compact scale, global normalization eliminates these absolute differences, which are essential for reliable defect classification.

The risk of overfitting is mitigated by the compact architecture of the models, with the transformer and CNN–transformer comprising 1.65 M and 1.86 M parameters, respectively. By processing low-dimensional sequences of four engineered features instead of high-dimensional raw pixel data, the networks are constrained to learn essential spatiotemporal interactions between physical descriptors. This reduction in both parameter space and input dimensionality ensures robust generalization and stable convergence despite the modest dataset size.

Approaches were assessed using mAP and standard deviation across validation folds for window/sequence lengths of 8, 16, 32, 64, 96, and 128. Per-class performance was evaluated using maximum F1-score, precision, and recall at optimized decision thresholds. All experiments were implemented in Python 3.10 with the PyTorch 2.6 and Timm 1.0.24 frameworks. Training was performed on an NVIDIA GeForce RTX 2060 GPU (6 GB), while inference speed was benchmarked on an Intel Core i5-11600K CPU (3.90 GHz). Classifiers were trained using the AdamW optimizer ($\mathrm{learning}\;\mathrm{rate}=1\times 10^{-4}$, no decay), batch size 64, and binary cross-entropy loss.

## 3. Results

To assess the applicability of the proposed DL approach, we quantified the statistical relationships between the extracted spatter features and the observed weld defects. As the Kolmogorov–Smirnov test indicated that the feature distributions were non-normal, we employed Spearman’s rank correlation analysis. Features were extracted and aggregated according to the method outlined in Section 2.3, utilizing a sliding window of 64 frames (Figure 4b). The resulting correlation coefficients are presented in Table 5, where coefficients with a p-value≤0.05 are marked with an asterisk (*) to denote statistical significance.

As shown in Table 5, the strongest correlations were observed for incomplete penetration ($h_{i}$) and shrinkage groove ($h_{g}$). For these defects, the most informative features were associated with spatter quantity and welding zone temperature, whereas features related to spatter area and spatter temperature showed weaker associations. However, the final classifiers achieved high predictive accuracy for sagging ($h_{s}$) and linear misalignment ($h_{m}$), suggesting that the full set of temporal features provides a richer detection signal than individual pairwise correlations alone. We performed additional ablation study to investigate the influence of each feature on the final classification precision.

Next, five DL pipelines (Methods; Figure 5) were trained and evaluated using multiple window/sequence lengths. The macro-averaged mAP and the corresponding parameter counts are summarized in Table 6.

**Figure 6:**
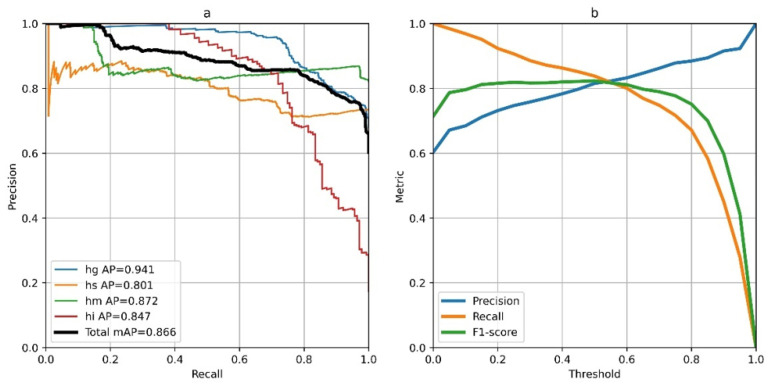
Evaluation curves of CNN–transformer: (**a**) precision–recall curves; (**b**) threshold–metric curves.

The results in Table 6 indicate that the hybrid CNN–transformer achieved the best overall performance, reaching an mAP of 0.85 at a sequence length of 64. This architecture also has the largest number of parameters among the tested models (1.86 M). In contrast, the simpler multi-head MLP, with substantially lower complexity (0.43 M parameters), delivered competitive performance (0.83 mAP), thereby offering a favorable efficiency–accuracy trade-off. Although the optimal sequence length differed across models, lengths between 32 and 96 frames generally provided the best balance between capturing sufficient temporal context and preserving an adequate number of training samples. Figure 6a shows the precision–recall curves of the best-performing CNN–transformer, aggregated across all validation folds and reported per defect class using micro-averaging. To select an operating point for deployment, we optimized the decision threshold by computing precision, recall, and the F1-score for thresholds from 0 to 1 in increments of 0.05. As shown in Figure 6b, the maximum F1-score occurred at a threshold of 0.55, which we therefore used as a general operating point. For specific industrial scenarios, this threshold can be adjusted to prioritize either higher precision or higher recall, depending on operational requirements.

We performed additional ablation study on feature importance to investigate whether the full set of temporal sequences could provide a richer detection signal than individual pairwise correlation presented in Table 5. During these experiments we trained and evaluated the proposed CNN–transformer as described in Section 2.4 using all possible feature combinations with a sequence length equal to 64 (the best result in Table 6). The ablation results are presented in Table 7 as per-class fold-wise Average Precision (AP) and total mAP for each feature combination sorted by mAP. Fold-wise standard deviation was omitted to make the results less cumbersome.

These results indicate strong correlation between spatter features and defects which is enough to classify defects based even on a single feature despite the relatively small pairwise correlation presented in Table 5 (especially for sagging and linear misalignment). Although single features achieved good performance (0.73–0.82 mAP), their right combinations produced better results (0.85 mAP for spatter area + number of spatters + spatter temp + welding zone temp). Notably, the temperature of the welding zone was the most informative feature as it outperformed some combinations of other features, whereas the spatter area was less informative. The combination of all features was used to produce the final results.

For comparison, we implemented two baseline models—MobileNetV4 Small [48] backbone with a two-layer linear classifier (1280 and 640 neurons) and 3D Convolutional ResNet-18 [39]—to compare against the proposed feature-based CNN–transformer pipeline. To ensure a fair comparison, both baselines processed thermograms at their native resolution (320×240 pixels) using the maximum batch size permitted by the hardware (64 for MobileNetV4 and 16 for 3D ResNet-18). Both baselines were trained using the Adam optimizer with binary cross-entropy loss, where the learning rate was set to $7\times 10^{-5}$ after being selected from a range of $10^{-3}$ to $10^{-6}$ with a step of 10 and no decay. Table 8 reports the AP, precision, recall, and F1-score for each defect class at the 0.55 decision threshold, presented as mean ± standard deviation across validation folds for the proposed model and both baselines. Table 8 also includes the mean processing speed per thermogram frame measured from the preprocessing start (the division on 255 for MobileNetV4 and 3D ResNet18, the pipeline in Figure 4a,d for the CNN–transformer) to the end of model’s inference over 1000 runs on the CPU.

The standard deviations across folds remain low for most defect classes, indicating overall model stability. However, the incomplete penetration ($h_{i}$) class exhibits significantly higher variability, suggesting that its detection performance is more sensitive to specific data splits.

Overall, the proposed time series pipeline based on the hybrid CNN–transformer provides a near-optimal balance between accuracy and computational efficiency, achieving 0.85 mAP (Table 8). It closely approaches the highest accuracy of the 3D ResNet-18 baseline (0.87 mAP) while delivering substantially faster inference (15.29 ms per frame), which supports real-time defect detection in pipeline welding. Moreover, the CNN–transformer attains the highest macro-averaged precision (0.84), indicating fewer false-positive predictions. By comparison, the lightweight MobileNetV4 baseline achieves the highest speed but yields the lowest overall accuracy (0.83 mAP). Consequently, the CNN–transformer is the most suitable architecture for the proposed pipeline, combining strong predictive performance with the responsiveness required for real-time monitoring.

## 4. Discussion

### 4.1. Interpretation of Key Findings

The findings of this study confirm the feasibility of detecting four defect types—incomplete penetration ($h_{i}$), shrinkage groove ($h_{g}$), sagging ($h_{s}$), and linear misalignment ($h_{m}$)—with the mAP of 0.85. This level of performance is achieved through the extraction of spatiotemporal features from thermogram sequences. While several defect types occur in fewer than 5% of cases, the use of macro-averaged metrics for model selection ensured that these rare classes contributed equally to the performance evaluation, preventing the model from simply ignoring them. The proposed pipeline integrates a thermogram processing algorithm (Figure 3 and Figure 4a) for generating feature time series, followed by the classification of fixed-length segments using a CNN–transformer architecture (Figure 4d and Figure 5e). Importantly, the low per-frame inference time of 15.3 ms on a CPU highlights the potential for real-time deployment in industrial environments, including implementation on edge devices such as Rockchip and NVIDIA Jetson. Such real-time feedback could support closed-loop control systems by enabling dynamic adjustment of laser parameters to reduce defect occurrence.

It should be acknowledged that the reported performance gap between the feature-based CNN–transformer and the end-to-end image-based baselines may be influenced by the depth of hyperparameter optimization. While more exhaustive tuning of architectures like MobileNetV4 could potentially close this gap, the primary focus of this study was to demonstrate that a compact set of physically informative features provides a robust and computationally efficient signal for defect detection. Unlike “black box” end-to-end models, our approach prioritizes physical interpretability and achieves high accuracy with significantly lower computational overhead for industrial real-time monitoring.

Additional insight comes from the per-class AP values in Table 8, reported as the mean and standard deviation across folds. The standard deviation serves as an indicator of model stability across different data splits. Notably, the incomplete penetration ($h_{i}$) defect exhibits a markedly higher standard deviation than the other classes. This variability is consistent across all tested architectures, suggesting it stems from characteristics of the dataset rather than a limitation of a specific model. In the current annotation strategy, a sample was labeled as defective when the $h_{i}$ depth exceeded zero according to ISO 6520-1. Experimental observations indicate that a higher physical defect magnitude may be required before it produces a consistently discernible thermal signature. Consequently, some validation folds contained sequences with weak or ambiguous signals, lowering the AP for those specific folds.

### 4.2. Comparative Analysis

The findings of this study confirm the feasibility of using spatiotemporal features as a reliable indicator for weld quality monitoring in pipeline steel FPLW. Our results align with the broader research trend toward automated defect detection, yet provide distinct advantages in terms of feature interpretability and computational efficiency. A distinction of our approach lies in the input data representation. In contrast to the methodology proposed by Zhang et al. [38], which utilizes a convolutional neural network for real-time penetration state diagnosis based on high-dimensional raw pixel sequences, our pipeline leverages a compact set of four physically interpretable engineered features. While Zhang et al. achieved a high accuracy of 94.6% for specific penetration states, our framework addresses the more complex task of multi-label classification, targeting four distinct defect types ($h_{i}$, $h_{g}$, $h_{s}$, and $h_{m}$) simultaneously and achieving a competitive macro-averaged mAP of 0.85. Compared to the feature extraction method by Gao et al. [37], which relies on HU moments of the weld pool and keyhole morphology to achieve 91.29% accuracy for six defect types, our hybrid CNN–transformer architecture explicitly models the temporal evolution of spatter ejections. This allows for a more robust interpretation of process instabilities compared to static geometric descriptors. Our achieved inference speed of 15.3 ms compares favorably with recent Vision Transformer models, such as those presented by Din et al. [42], which report 97% accuracy for battery pole defects but require substantially larger datasets and more complex attention mechanisms to operate on raw images.

### 4.3. Limitations and Challenges

As demonstrated, statistically significant relationships between extracted features and weld defects were identified only for four defect types. The current set of extractable features is constrained by the spatial, temporal, and thermal properties of the spatters, which inherently limits the number of detectable welding anomalies.

A further challenge concerns the resolution of the thermal imaging sensor, which can impede the accurate detection of small spatters or introduce errors in estimating their size and temperature. Data availability remains a significant constraint—the dataset comprises 34 thermograms, yielding 800 samples with a window size of 64 and a 50% overlap. Furthermore, the industrial focus of this study was primarily on incomplete penetration ($h_{i}$), other defect types were included and reported to demonstrate the framework’s potential for multi-label classification in broader welding despite the pronounced class imbalance. Moreover, the current study did not assess the impact of different materials, camera angles, or acquisition conditions, all of which may influence feature extraction and classifier performance.

### 4.4. Future Research Directions

Building on the findings of this study, future work should focus on the development of a substantially larger and more diverse dataset, incorporating diverse sets of materials, welding conditions, and thermogram acquisition configurations. Such a dataset would enable a more rigorous assessment of the generalizability and robustness of the proposed methodology. To enhance measurement precision, future studies should also explore the use of thermal imaging systems with higher spatial resolution and increased frame rates.

Future work will focus on enriching standard deep learning architectures with physics-informed inductive biases, including specialized attention mechanisms and tailored loss functions that reflect the underlying welding dynamics. Beyond the evaluated architectures, we plan to investigate hierarchical temporal models and multi-modal transformers to capture the evolution of ejections across multiple spatiotemporal scales. These developments will be complemented by the integration of domain-specific knowledge—such as temperature decay patterns and the transient nature of spatter ejections—into both architectural design and optimization objectives, thereby enhancing detection sensitivity and predictive accuracy. At the system level, further performance gains are expected through advanced classifier refinement and sensor fusion strategies that combine thermal imaging with visible and ultraviolet imaging, spectroscopy, or other auxiliary sensing modalities. Ultimately, such sensor fusion will allow for a more dependable way to detect various defects in real time, making industrial laser welding significantly more reliable.

Further development plans for this approach include its integration into a multi-agent system to enhance automation of engineering workflows and process control, while improving usability through natural-language interaction.

## 5. Conclusions

This work establishes a sensor-to-AI framework for non-destructive, in-process defect detection in full-penetration laser welding using infrared thermography as the primary monitoring modality. A dedicated thermogram-processing stage isolates spatter activity and converts high-speed IR video into compact, physically interpretable time series descriptors (spatter area, spatter temperature, number of spatters, and welding zone temperature), enabling data-driven quality assessment directly from sensor signals.

A multi-label learning formulation was adopted to reflect real manufacturing conditions where multiple defects may co-occur, targeting incomplete penetration, sagging, shrinkage groove, and linear misalignment. Across the evaluated model families, the hybrid CNN–transformer that processes fixed-length feature sequences delivered the strongest overall performance, achieving 0.85 mAP at a sequence length of 64 frames, while maintaining near real-time inference (15.3 ms per frame on the CPU), which supports practical deployment without specialized compute.

This work establishes a robust real-time defect detection framework based on the first systematic evaluation of the relationships between multi-dimensional spatter dynamics and diverse weld defects in pipeline steel. Particularly strong results were achieved for incomplete penetration defects, where the correlation between spatter signatures and defect formation proved to be highly discriminative in real time.

By leveraging non-specialized thermal cameras rather than high-cost proprietary hardware from Precitec, Trumpf, or IPG, the proposed methodology provides a scalable and economically viable foundation for automated in-process quality assurance.

These findings demonstrate that for 09G2S pipeline steel, IR thermography coupled with temporal deep learning on engineered spatiotemporal features can provide actionable online diagnostics under laboratory conditions. While industrial deployment requires further testing across diverse setups, this work provides a preliminary foundation for the potential development of in-process parameter optimization systems in industrial pipeline laser welding.

## Figures and Tables

**Table 1:** Chemical composition (wt., %) and mechanical properties of 09G2S grate steel according to GOST 19281-2014 [45].

**C**	**Si**	**Mn**	**Ni**	**S**	**P**	**Cr**	**V**	**N**	**Cu**	**As**
<0.12	0.5–0.8	1.3–1.7	<0.3	<0.035	<0.03	<0.3	<0.12	<0.008	<0.3	<0.08
**Tensile strength (MPa)**					**Elongation (%)**					
≥490					≥21					

**Table 2:** Specification of experimental setup.

**MAXphotonics MFSC-3000**	**Units**	**Levels**
Nominal output power	W	3000
Optical fibre diameter	μm	100
Spot diameter at focus	mm	0.14
**Welding optics: WSX ND40**		
Collimating length	mm	100
Focusing length	mm	250
Emission wavelength	nm	1080
**FLIR Systems CEDIP TITANIUM 520 M Camera**		
Resolution	pixel	320 × 240
Frequency	Hz	380
Spectral range	μm	1.5–5.0

**Table 3:** Experimental parameters, factors, and levels.

**Constant Welding Parameter**		**Value**	
$F_{z}$, mm		0	
α, °		15	
**Factors and levels**			
**Factor**	**Level 1**	**Level 2**	**Level 3**
$P_{L}$, W	1500	2000	2500
$V_{w}$, mm/s	30	35	40

**Table 4:** Investigated welding defects and their occurrence in the experimental set.

Defect Designation	ISO 6520-1 [46]	ID	Occurr.	Illustration
Incomplete penetration	402	$h_{i}$	17%	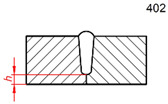
Linear misalignment	507	$h_{m}$	3%	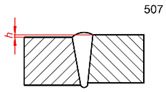
Sagging	509	$h_{s}$	5%	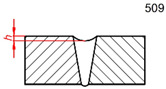
Shrinkage groove	515	$h_{g}$	1%	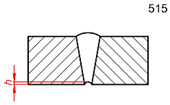

**Table 5:** Spearman’s feature–defect correlation coefficients and *p*-values.

Defect	Size Mean	Size Std	Number of Spatters Mean	Number of Spatters Std	Spatter Temp Mean	Spatter Temp Std	Welding Zone Temp Mean	Welding Zone Temp Std
$h_{g}$	0.08	−0.09	0.22 *	0.20 *	0.00	−0.27 *	−0.56 *	−0.39 *
$h_{s}$	−0.01	0.01	0.04	0.09	0.03	−0.03	−0.19 *	−0.07
$h_{m}$	0.16 *	0.06	0.00	−0.05	0.14 *	−0.14 *	−0.05	0.02
$h_{i}$	−0.35 *	0.10	−0.43 *	−0.40 *	−0.28 *	0.37 *	0.59 *	0.50 *

**Table 6:** Mean Average Precision of Deep Learning Approaches.

Model	Sequence Size					
	8	16	32	64	96	128
Multi-Head MLP (0.43 M)	0.80±0.06	0.82±0.06	0.83±0.05	0.83±0.07	0.83±0.07	0.83±0.06
CNN (0.44 M)	0.79±0.07	0.81±0.08	0.81±0.07	0.82±0.07	0.78±0.03	0.81±0.08
LSTM (1.32 M)	0.80±0.06	0.81±0.06	0.81±0.06	0.82±0.05	0.83±0.05	0.82±0.09
Transformer (1.65 M)	0.79±0.05	0.78±0.05	0.80±0.04	0.78±0.05	0.78±0.06	0.77±0.05
CNN–Transformer (1.86 M)	0.81±0.07	0.82±0.07	0.82±0.06	0.85±0.04	0.82±0.06	0.83±0.07

The bold font highlights the best results in the table.

**Table 7:** Ablation study on the feature importance.

Feature Combination	AP				mAP
	$h_{g}$	$h_{s}$	$h_{m}$	$h_{i}$	
spatter area + number of spatters + spatter temp + welding zone temp	0.94	0.85	0.87	0.72	0.85
number of spatters + spatter temp + welding zone temp	0.95	0.78	0.84	0.78	0.84
spatter area + spatter temp + welding zone temp	0.95	0.80	0.85	0.71	0.83
spatter temp + welding zone temp	0.94	0.83	0.86	0.68	0.83
spatter area + number of spatters + welding zone temp	0.94	0.78	0.88	0.71	0.83
number of spatters + welding zone temp	0.94	0.77	0.88	0.71	0.83
welding zone temp	0.95	0.76	0.82	0.76	0.82
spatter area + welding zone temp	0.94	0.75	0.86	0.73	0.82
number of spatters + spatter temp	0.83	0.74	0.87	0.56	0.75
spatter area + number of spatters + spatter temp	0.85	0.75	0.90	0.50	0.75
spatter area + number of spatters	0.84	0.71	0.89	0.55	0.75
number of spatters	0.81	0.73	0.88	0.56	0.75
spatter area + spatter temp	0.80	0.76	0.86	0.55	0.74
spatter temp	0.80	0.74	0.86	0.54	0.73
spatter area	0.75	0.74	0.88	0.54	0.73

**Table 8:** Per-class evaluation results.

Model	Metric	Classes				Macro Avg	Speed ms
		$h_{g}$	$h_{s}$	$h_{m}$	$h_{i}$		
CNN– Transformer (1.86 M)	AP	0.94±0.02	0.86±0.14	0.87±0.09	0.72±0.15	0.85	15.29
	Precision	0.87±0.07	0.73±0.20	0.87±0.05	0.88±0.13	0.84	
	Recall	0.81±0.06	0.81±0.14	0.97±0.04	0.62±0.30	0.80	
	F1-score	0.83±0.01	0.74±0.11	0.92±0.02	0.67±0.26	0.79	
MobileNetV4 (1.78 M)	AP	0.95±0.03	0.84±0.09	0.80±0.09	0.75±0.38	0.83	6.49
	Precision	0.90±0.06	0.82±0.12	0.84±0.08	0.70±0.34	0.81	
	Recall	0.87±0.07	0.85±0.10	0.83±0.11	0.76±0.17	0.83	
	F1-score	0.88±0.06	0.83±0.10	0.83±0.08	0.70±0.30	0.81	
3D ResNet18 (33.16 M)	AP	0.95±0.03	0.93±0.05	0.87±0.05	0.73±0.33	**0.87**	237.90
	Precision	0.89±0.08	0.84±0.10	0.85±0.07	0.73±0.28	0.83	
	Recall	0.88±0.04	0.92±0.06	0.89±0.06	0.67±0.30	0.84	
	F1-score	0.88±0.04	0.87±0.06	0.87±0.06	0.69±0.28	0.83	

## Data Availability

The data and source code supporting the findings of this study can be downloaded from: https://github.com/ILT-ITMO/LaserWeldMonitor (accessed on 9 March 2026).

## Abbreviations

The following abbreviations are used in this manuscript:

FPLW	Full-Penetration Laser Welding
IR	Infrared
LBW	Laser Beam Welding
GMAW	Gas Metal Arc Welding
SAW	Submerged Arc Welding
WPDC	Weld Penetration Depth Control
ML	Machine Learning
DT	Decision Tree
RF	Random Forest
RL	Logistic Regression
GB	Gradient Boosting
SVM	Support Vector Machine
DL	Deep Learning
MLP	Multilayer Perceptron
CNN	Convolutional Neural Networks
LSTM	Long Short-Term Memory
mAP	Mean Average Precision
LoG	Laplacian-of-Gaussian
$h_{g}$	Shrinkage Groove
$h_{s}$	Sagging
$h_{m}$	Linear Misalignment
$h_{i}$	Incomplete Penetration
AP	Average Precision
